# Supplementary figures and images for: Divergent resistance pathways amongst SARS-CoV-2 PLpro inhibitors highlight the need for scaffold diversity
Source: PLoS Pathog. 2025 Sep 5;21(9):e1013468. doi: 10.1371/journal.ppat.1013468 (PMC12431669; doi:10.1371/journal.ppat.1013468)

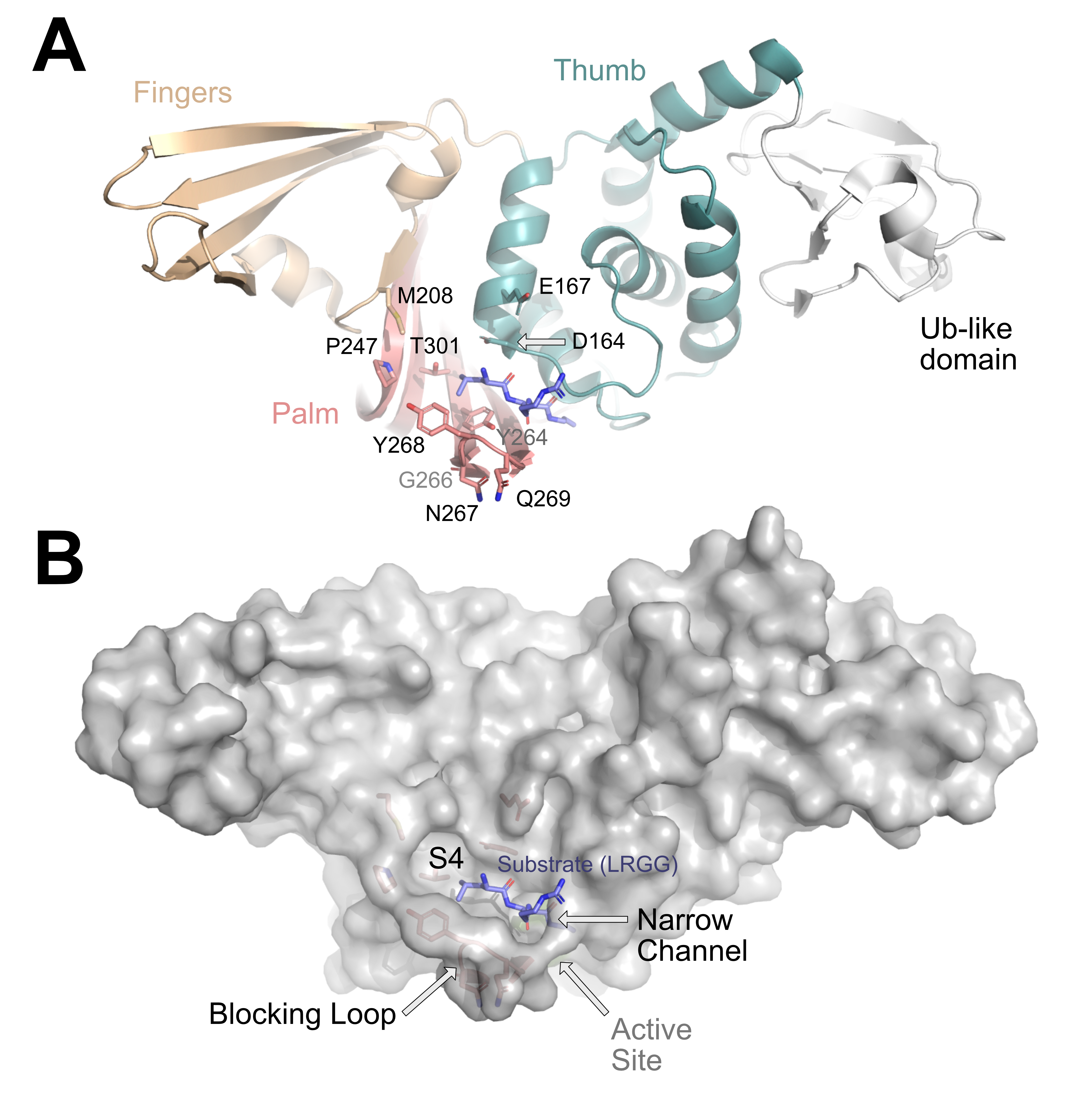

Supplement: S1 Fig — A-B) structural representation of PLpro bound to LRGG, which represents the final four residues at the C-terminus of ubiquitin (PDB: 6XAA) [14]. A) highlights domains: the ubiquitin-like domain in grey, thumb domain in cyan, palm domain in red, and fingers domain in orange. The LRGG substrate is depicted in blue, with its key interacting residues shown in stick representation. B) presents PLpro in a surface view, highlighting the S4 pocket, blocking loop, active site and the narrow channel leading to the active site. (TIFF) [file ppat.1013468.s001.tiff]

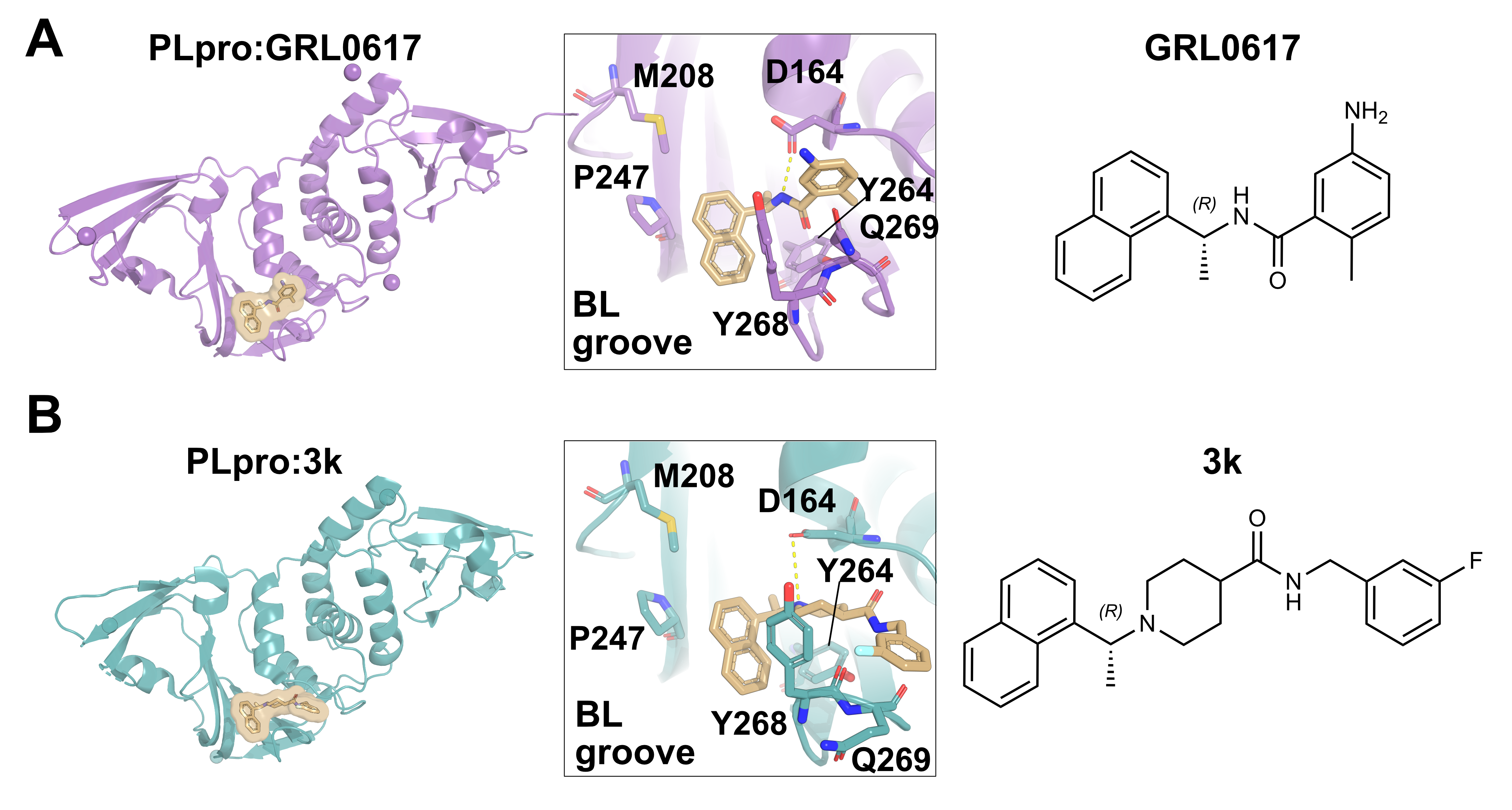

Supplement: S2 Fig — A-B) structural representation of PLpro bound to three inhibitors GRL0617 (PDB: 7CJM, PLpro in purple) [16], and 3k (PDB: 7TZJ, PLpro in green) [15]. The central panel zooms in on these compounds and their surroundings, highlighting key interaction partners. The right panel displays the structural formulae of these compounds. (TIFF) [file ppat.1013468.s002.tiff]

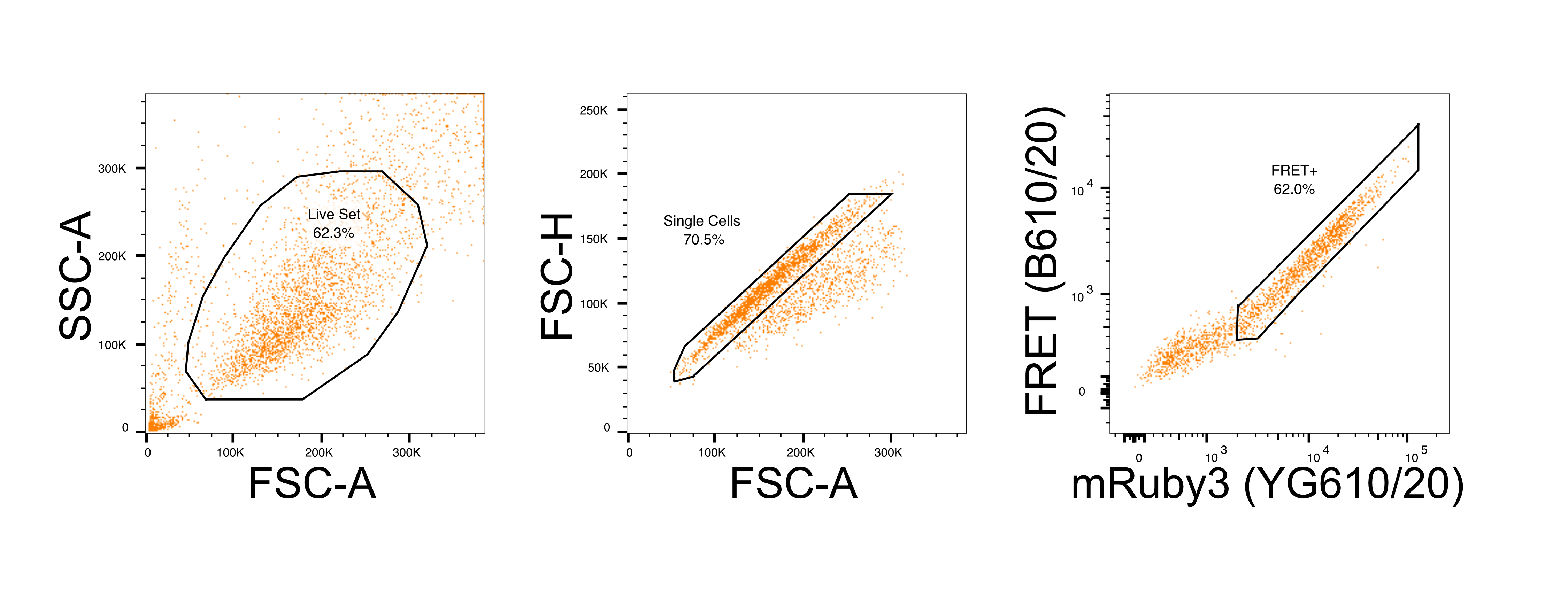

Supplement: S3 Fig — Live cells are identified using forward-scatter (FSC-A) and side scatter (SSC-A) parameters. Single cells were isolated by comparing FSC-A and FSC-H. FRET positive cells were detected using mRuby3 fluorescence (YG610/20) and FRET (B610/20). (TIFF) [file ppat.1013468.s003.tiff]

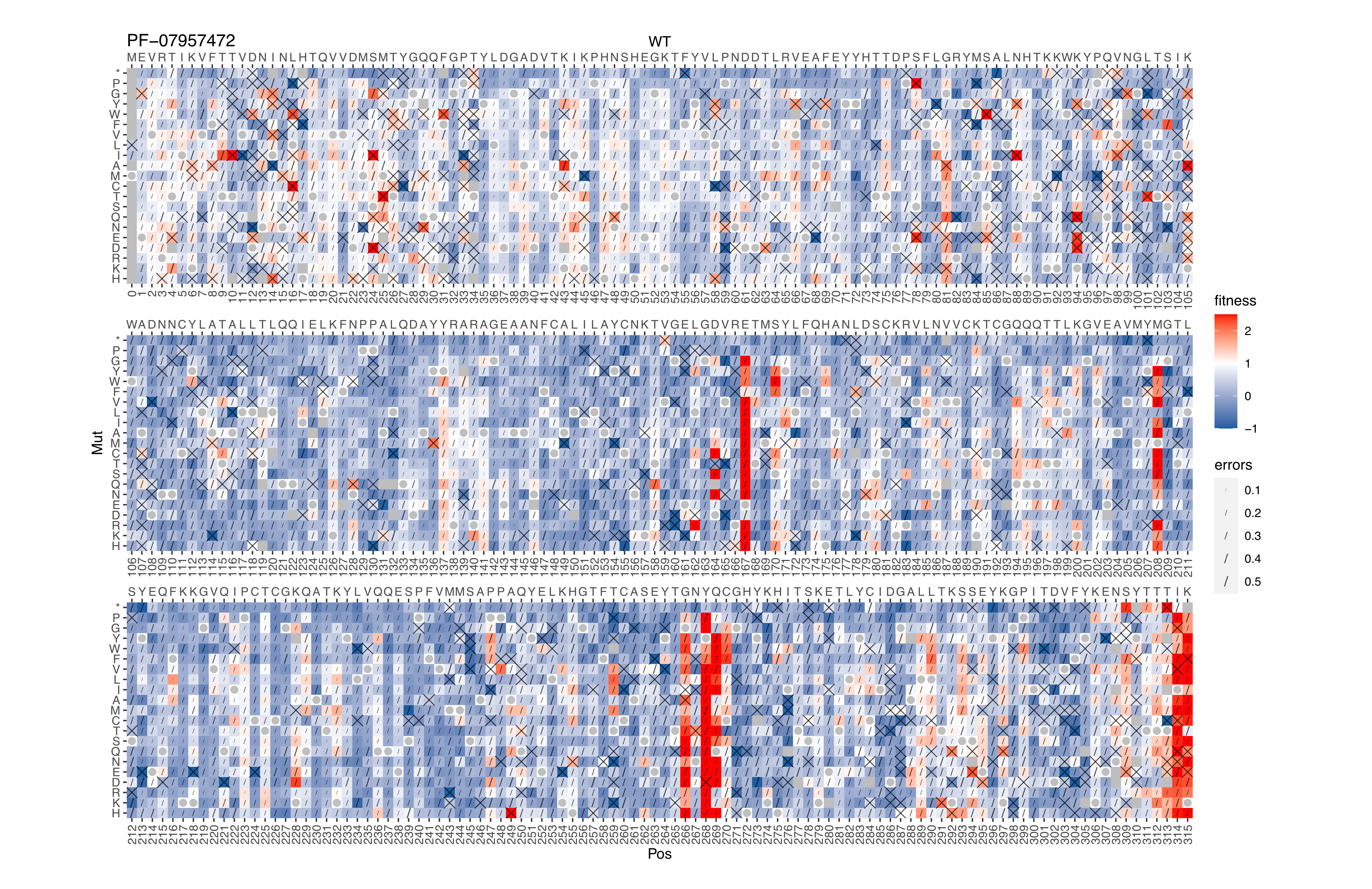

Supplement: S4 Fig — This sequence-function map shows normalized DiMSum fitness scores calculated from FRET+ versus FRET- gates after inhibitor treatment. Variants are arranged with residue number on the x-axis and mutation type on the y-axis. Fitness scores were normalized so that the mean of wildtype and nonsense variants (1–305) are 1 and 0 respectively. The color scale represents the normalized fitness scores for each variant. Each square corresponds to a single-residue substitution and includes an inset slash whose length is proportional to the estimated error. Wildtype residues are highlighted with a solid circle, and variants with an error greater than 0.5 are marked with a cross. (TIFF) [file ppat.1013468.s004.tiff]

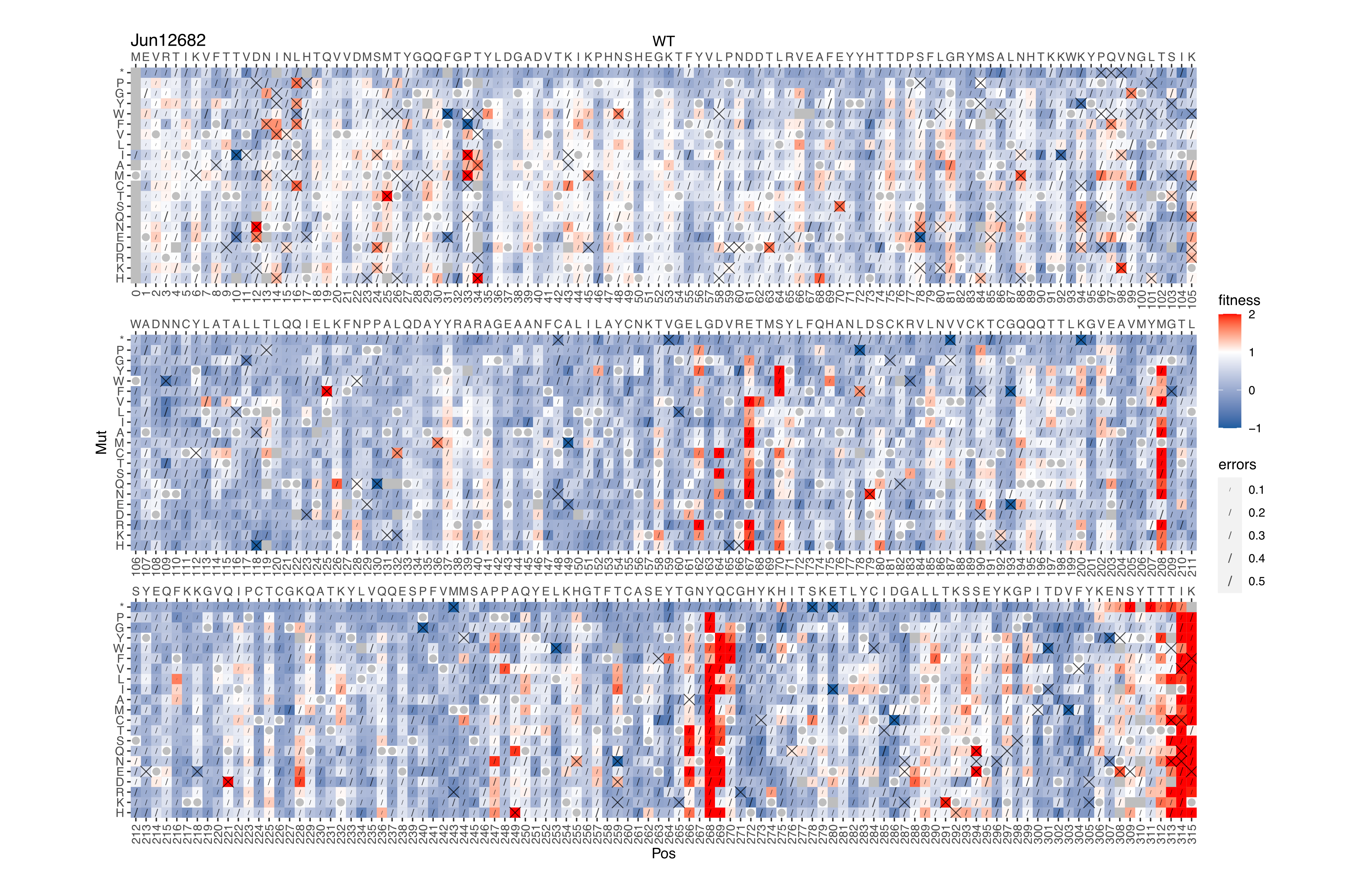

Supplement: S5 Fig — This sequence-function map shows normalized DiMSum fitness scores calculated from FRET+ versus FRET- gates after inhibitor treatment. Variants are arranged with residue number on the x-axis and mutation type on the y-axis. Fitness scores were normalized so that the mean of wildtype and nonsense variants (1–305) are 1 and 0 respectively. The color scale represents the normalized fitness scores for each variant. Each square corresponds to a single-residue substitution and includes an inset slash whose length is proportional to the estimated error. Wildtype residues are highlighted with a solid circle, and variants with an error greater than 0.5 are marked with a cross. (TIFF) [file ppat.1013468.s005.tiff]

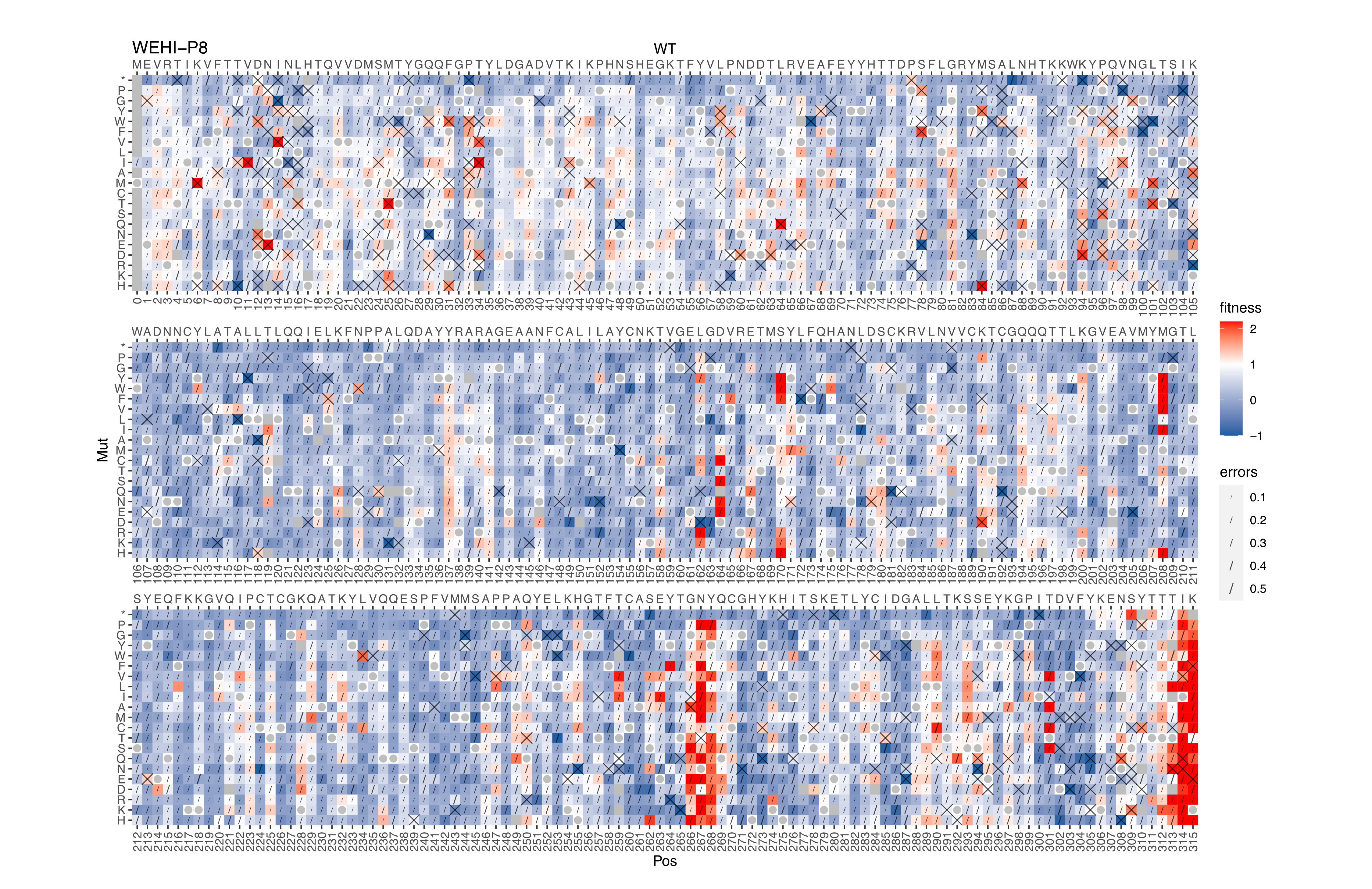

Supplement: S6 Fig — This sequence-function map shows normalized DiMSum fitness scores calculated from FRET+ versus FRET- gates after inhibitor treatment. Variants are arranged with residue number on the x-axis and mutation type on the y-axis. Fitness scores were normalized so that the mean of wildtype and nonsense variants (1–305) are 1 and 0 respectively. The color scale represents the normalized fitness scores for each variant. Each square corresponds to a single-residue substitution and includes an inset slash whose length is proportional to the estimated error. Wildtype residues are highlighted with a solid circle, and variants with an error greater than 0.5 are marked with a cross. (TIFF) [file ppat.1013468.s006.tiff]

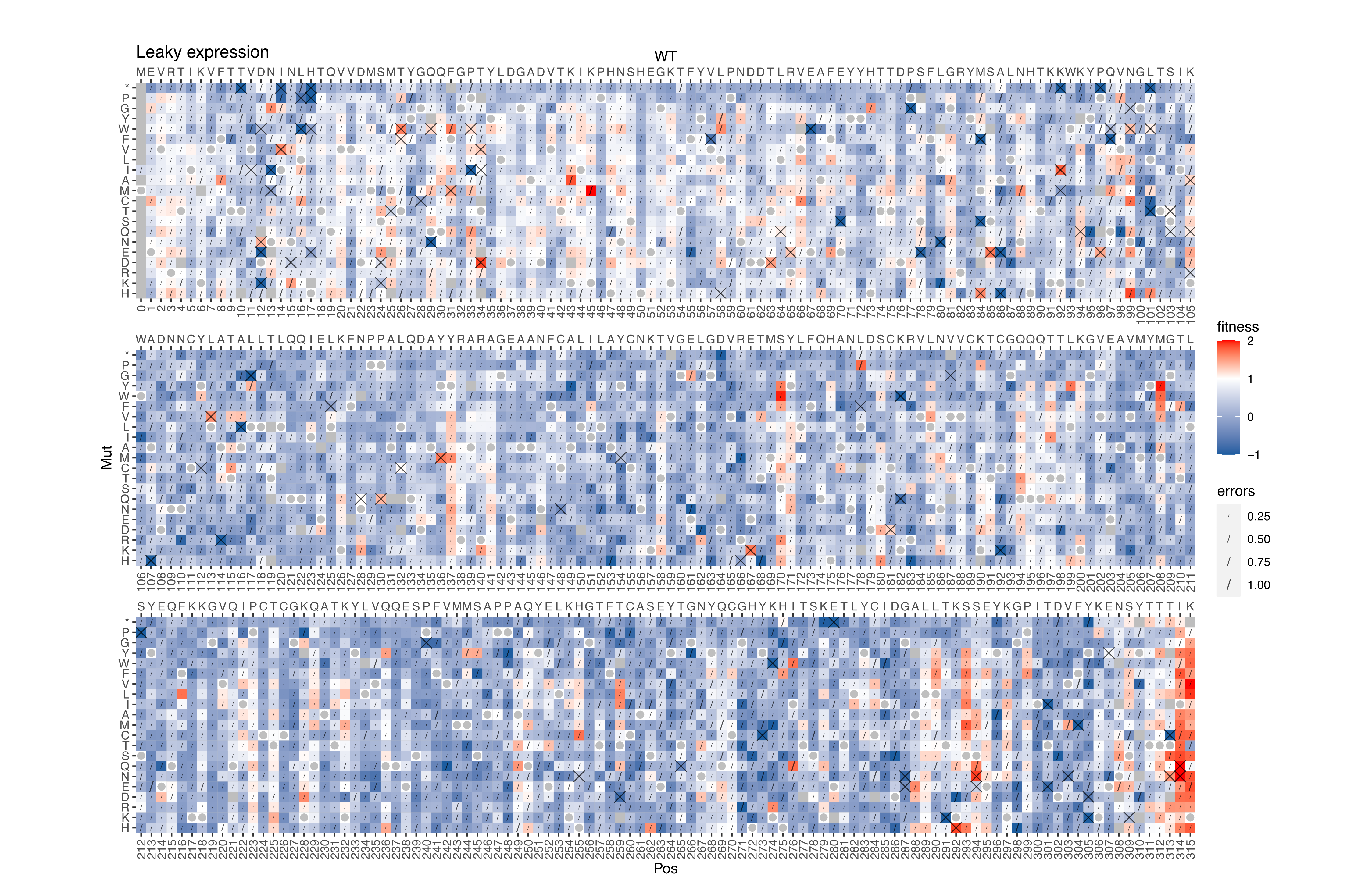

Supplement: S7 Fig — This sequence-function map shows normalized DiMSum fitness scores calculated from FRET+ versus FRET- gates prior to induction of PLpro expression by doxycycline. Variants are arranged with residue number on the x-axis and mutation type on the y-axis. Fitness scores were normalized so that the mean of wildtype and nonsense variants (1–305) are 1 and 0 respectively. The color scale represents the normalized fitness scores for each variant. Each square corresponds to a single-residue substitution and includes an inset slash whose length is proportional to the estimated error. Wildtype residues are highlighted with a solid circle, and variants with an error greater than 0.5 are marked with a cross. (TIFF) [file ppat.1013468.s007.tiff]

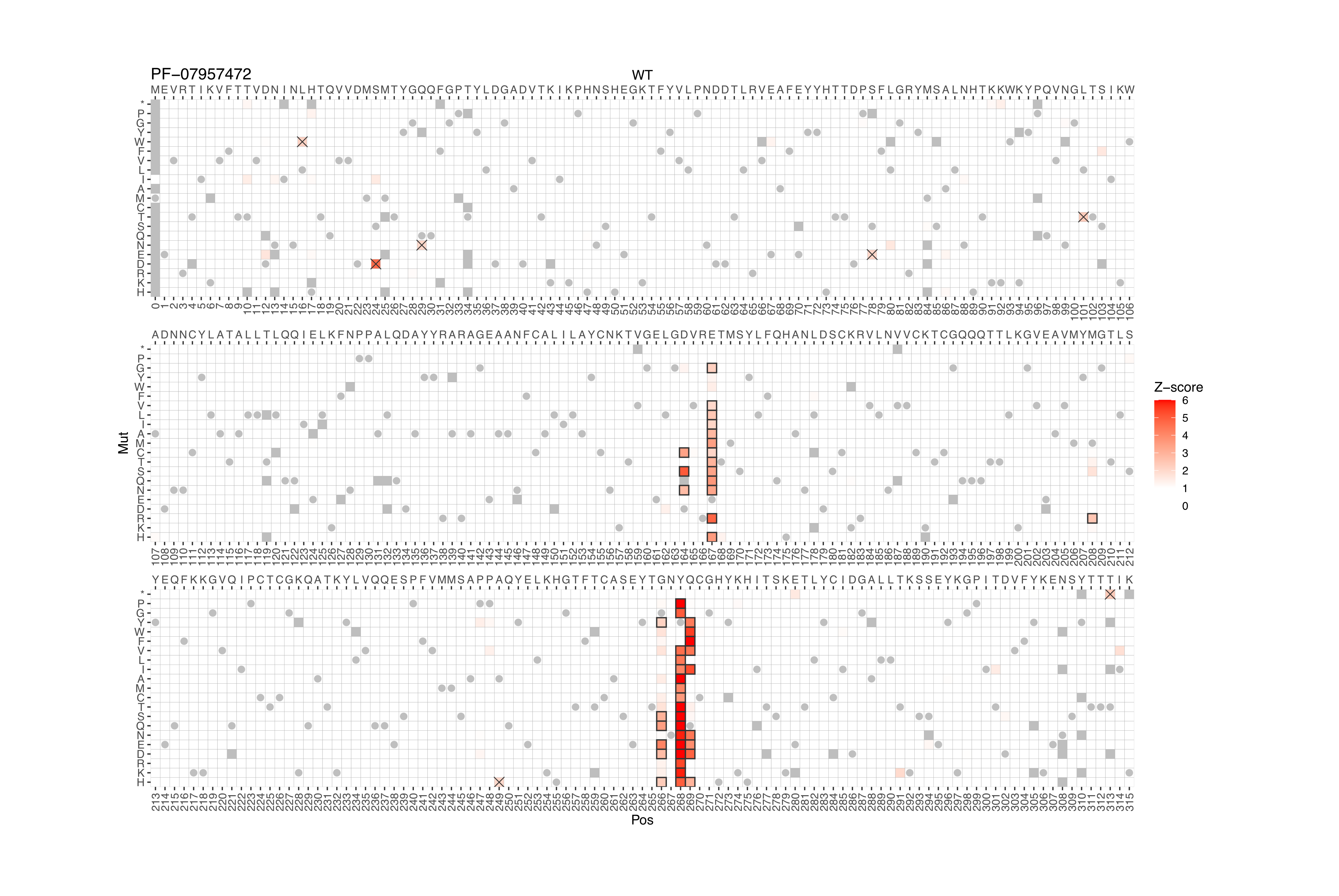

Supplement: S8 Fig — The sequence-function map displays Z-scores that have been corrected based on each variant’s activity and leaky expression scores and indicate the escape of each variant from inhibition. This data is also plotted in a different format in Fig 2B. Variants are arranged with residue number on the x-axis and mutation type on the y-axis. The color scale represents the Z-scores for each variant, with Z-scores less than 1 appearing white and Z-scores of more than 1 increasingly red. Each square corresponds to a single-residue substitution. Wildtype residues are highlighted with a solid circle. Variants that meet the filtering criteria from S20 Fig and have Z-scores higher than 2 are highlighted with boxes and used in the Venn diagrams depicted in Fig 4F and 4G. Variants with Z-scores over 2 but have low underlying counts or high errors in the contributing datasets are marked with a X. More information on these criteria can be found in the Methods section and S20 Fig. (TIFF) [file ppat.1013468.s008.tiff]

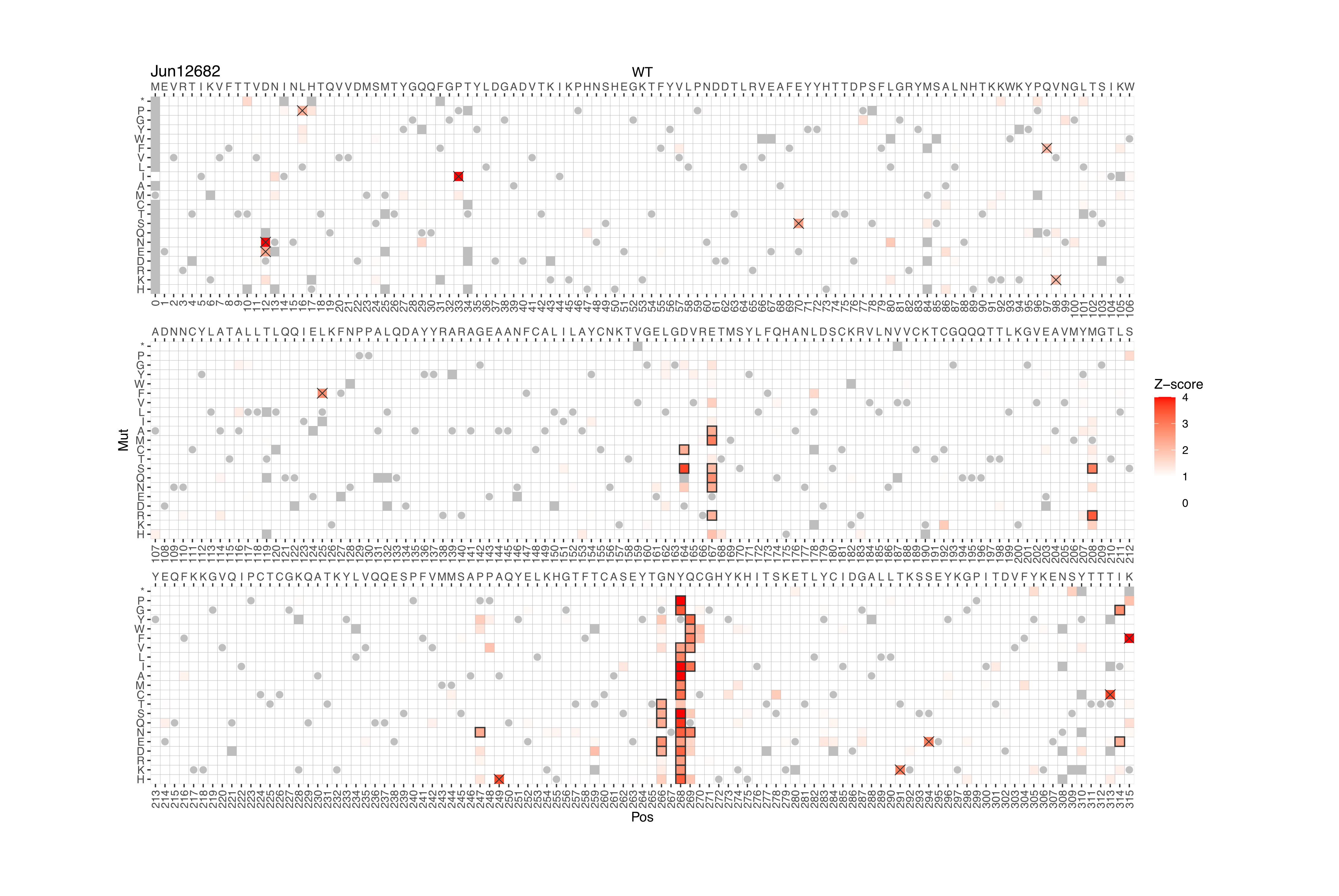

Supplement: S9 Fig — The sequence-function map displays Z-scores that have been corrected based on each variant’s activity and leaky expression scores and indicate the escape of each variant from inhibition. This data is also plotted in a different format in Fig 2B. Variants are arranged with residue number on the x-axis and mutation type on the y-axis. The color scale represents the Z-scores for each variant, with Z-scores less than 1 appearing white and Z-scores of more than 1 increasingly red. Each square corresponds to a single-residue substitution. Wildtype residues are highlighted with a solid circle. Variants that meet the filtering criteria from S20 Fig and have Z-scores higher than 2 are highlighted with boxes and used in the Venn diagrams depicted in Fig 4F and 4G. Variants with Z-scores over 2 but have low underlying counts or high errors in the contributing datasets are marked with a X. More information on these criteria can be found in the Methods section and S20 Fig. (TIFF) [file ppat.1013468.s009.tiff]

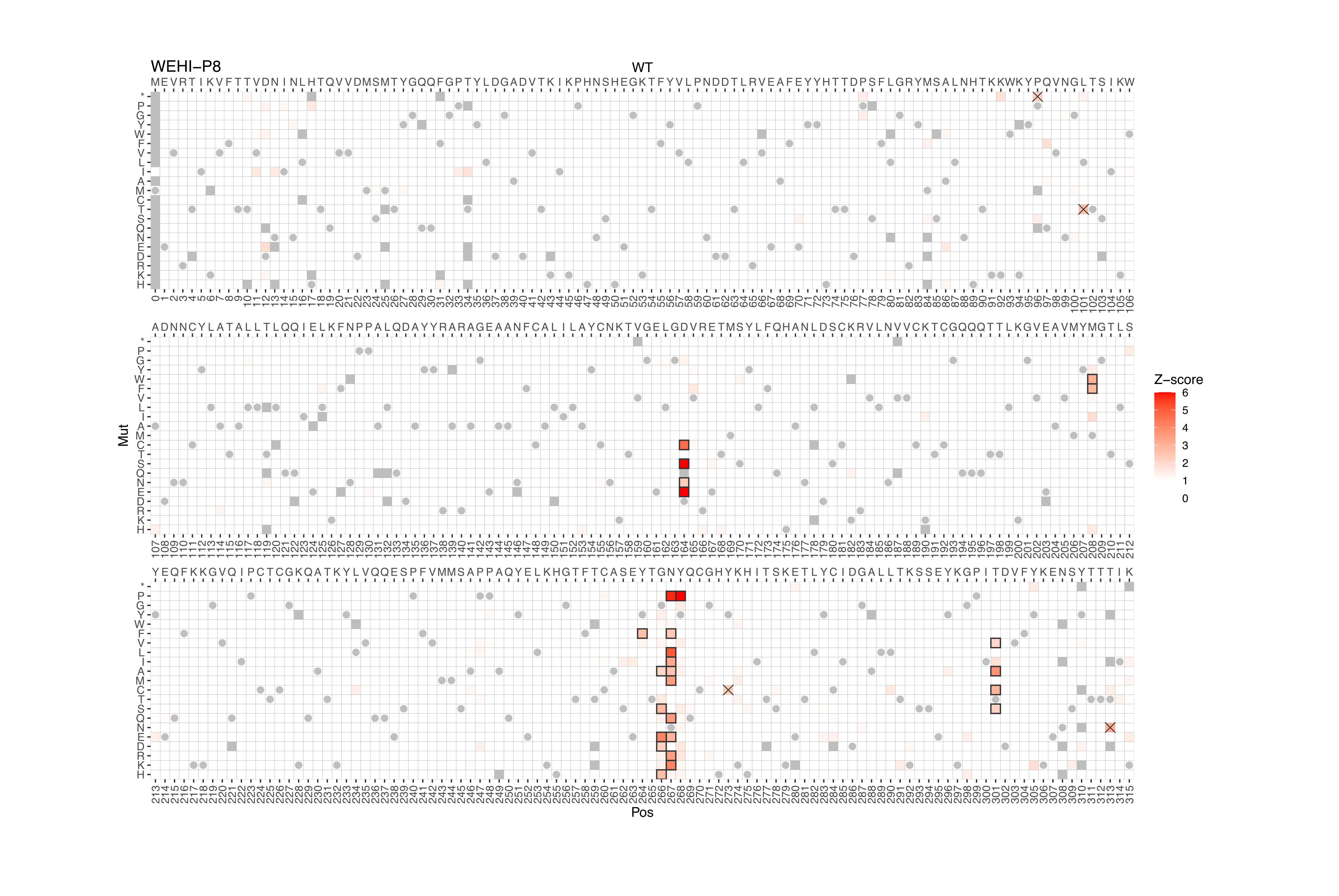

Supplement: S10 Fig — The sequence-function map displays Z-scores that have been corrected based on each variant’s activity and leaky expression scores and indicate the escape of each variant from inhibition. This data is also plotted in a different format in Fig 2B. Variants are arranged with residue number on the x-axis and mutation type on the y-axis. The color scale represents the Z-scores for each variant, with Z-scores less than 1 appearing white and Z-scores of more than 1 increasingly red. Each square corresponds to a single-residue substitution. Wildtype residues are highlighted with a solid circle. Variants that meet the filtering criteria from S20 Fig and have Z-scores higher than 2 are highlighted with boxes and used in the Venn diagrams depicted in Fig 4F and 4G. Variants with Z-scores over 2 but have low underlying counts or high errors in the contributing datasets are marked with a X. More information on these criteria can be found in the Methods section and S20 Fig. (TIFF) [file ppat.1013468.s010.tiff]

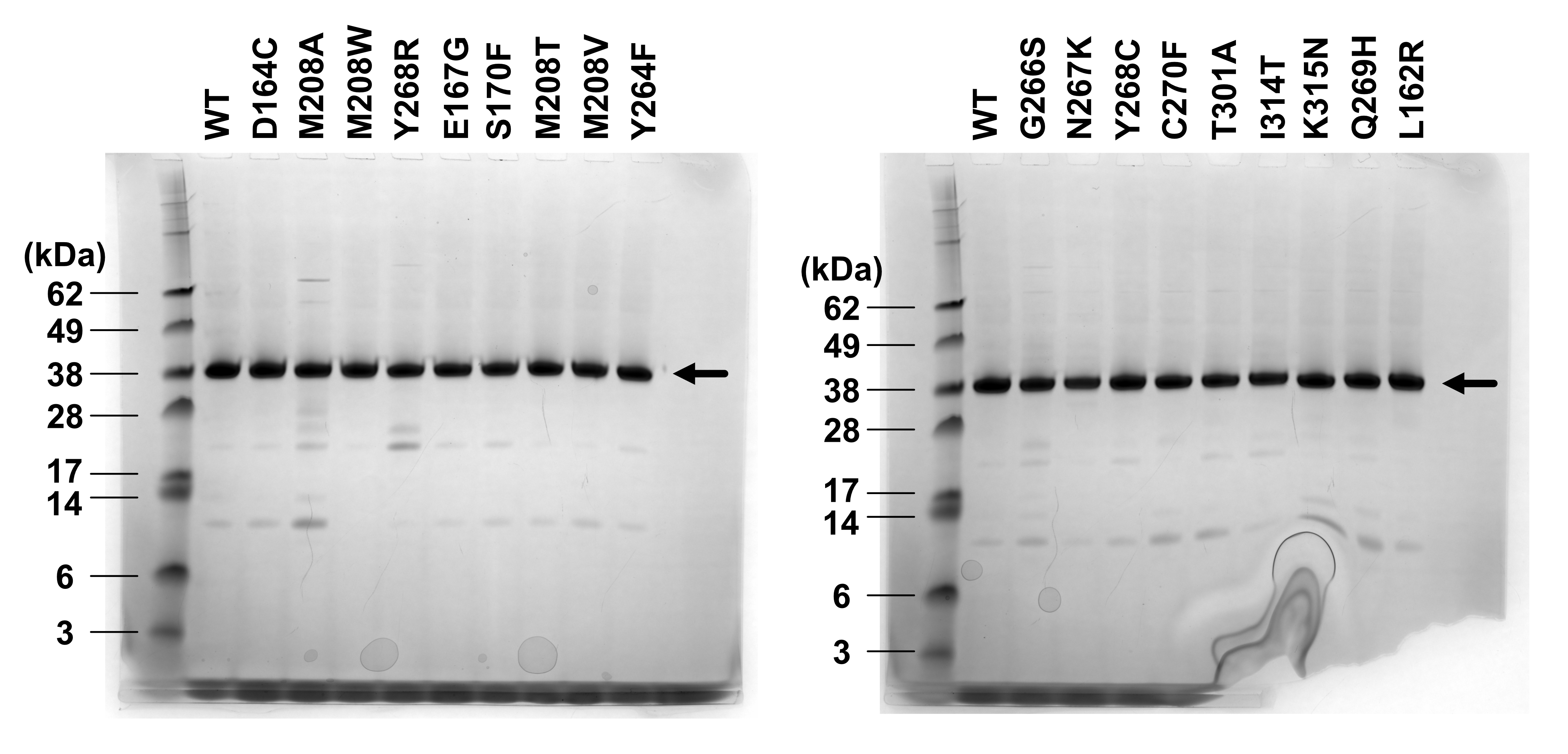

Supplement: S11 Fig — Recombinantly produced PLpro variants were run by SDS-PAGE to determine purity and relative concentration. The SeeBlue Plus2 pre-stained protein marker was used for molecular weight determination. The band with the expected molecular weight of PLpro is marked with an arrow. Each lane shows the respective PLpro variant as labeled, while the arrow indicates PLpro band. (TIFF) [file ppat.1013468.s011.tiff]

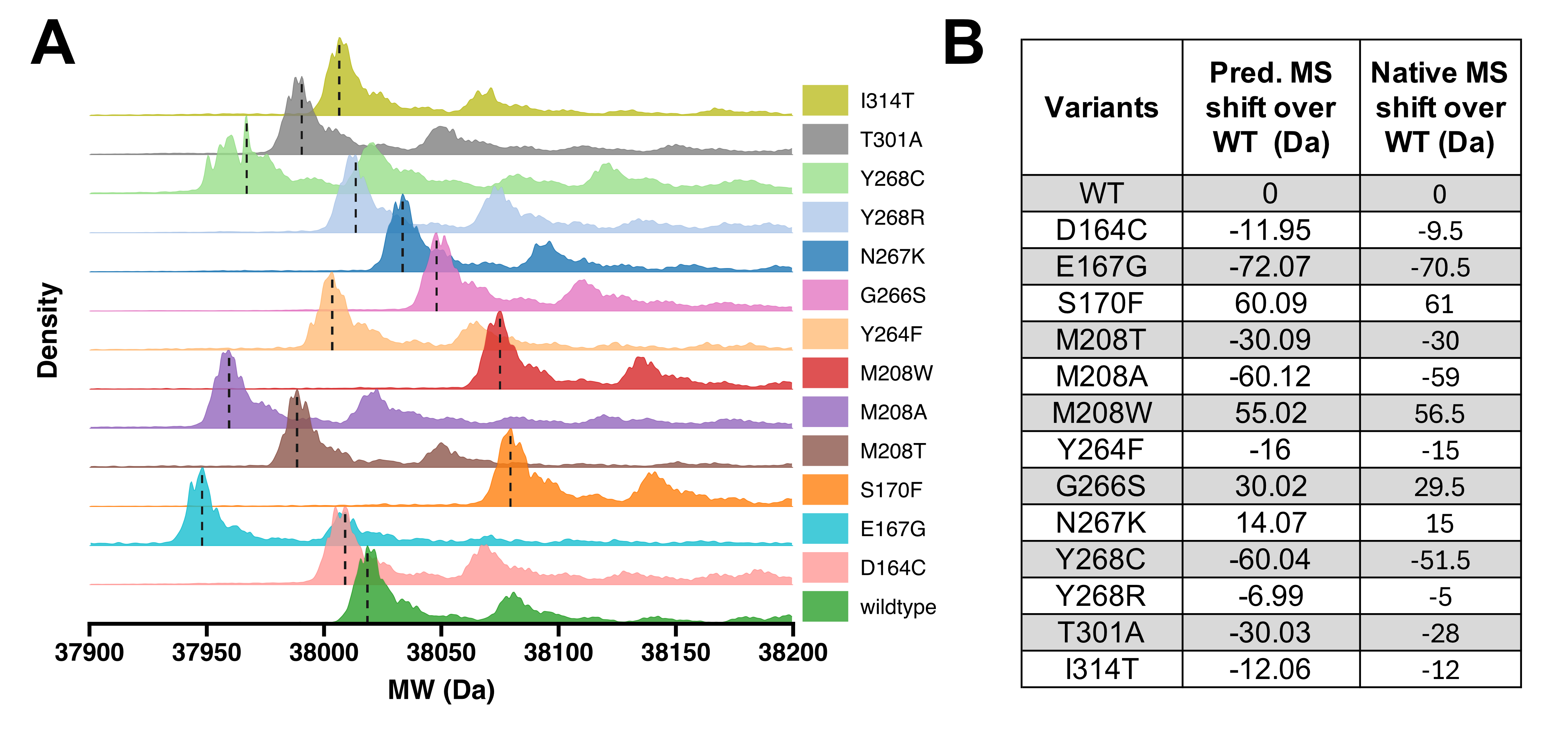

Supplement: S12 Fig — A) Deconvoluted spectra from native MS experiment are shown for selected PLpro variants. The primary peak – representing the variant’s mass – is highlighted with a dashed line and normalized to 100% intensity for clarity. B) The predicted versus observed mass shift of each variant from wildtype. (TIFF) [file ppat.1013468.s012.tiff]

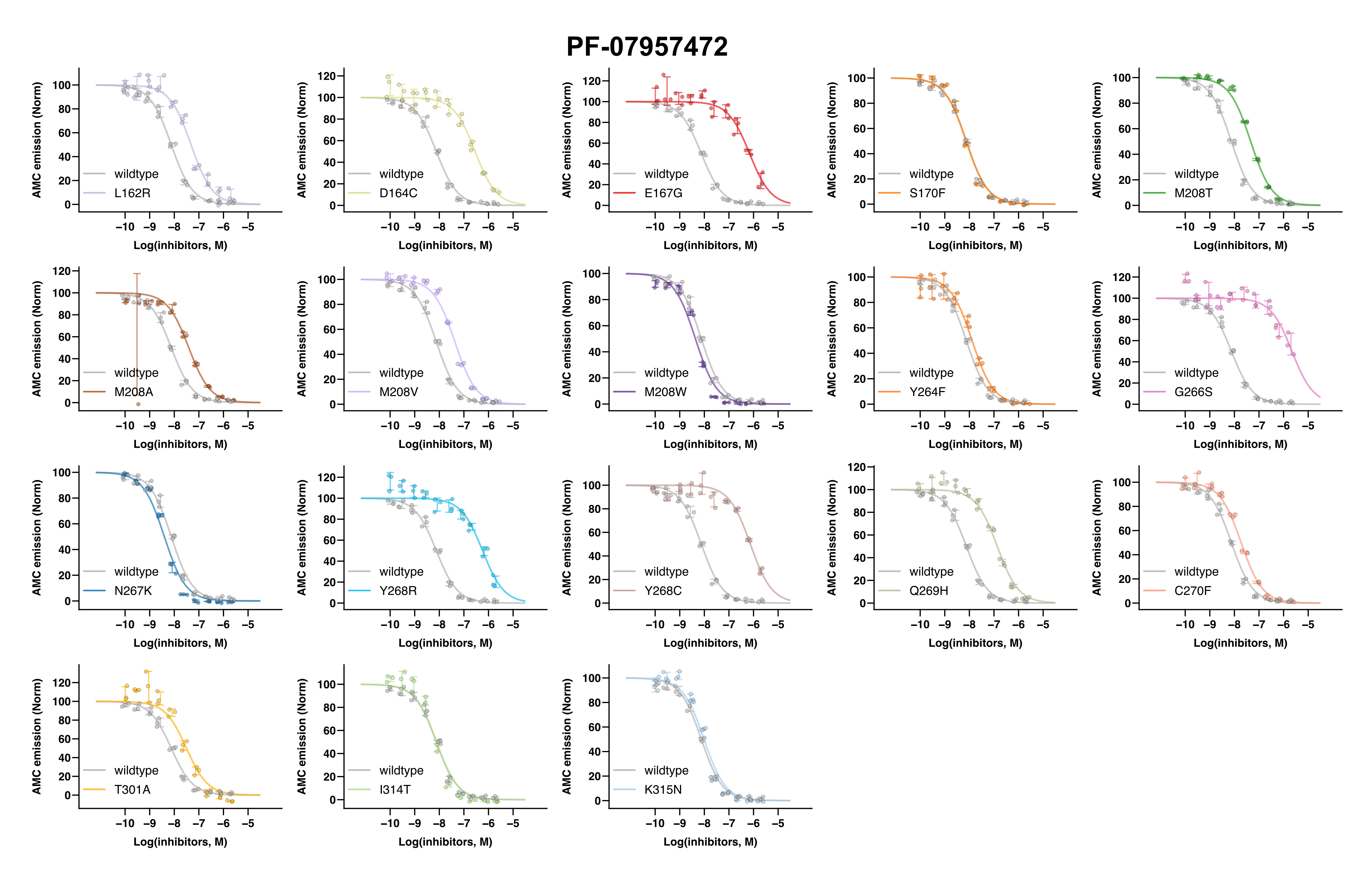

Supplement: S13 Fig — In each panel, the emission from buffer-only control (no PLpro) was normalized 0, and the emission from samples without inhibitor was normalized to 100. Inhibitor concentrations are plotted on a logarithmic scale on the x-axis, with normalized emission on the y-axis. (TIFF) [file ppat.1013468.s013.tiff]

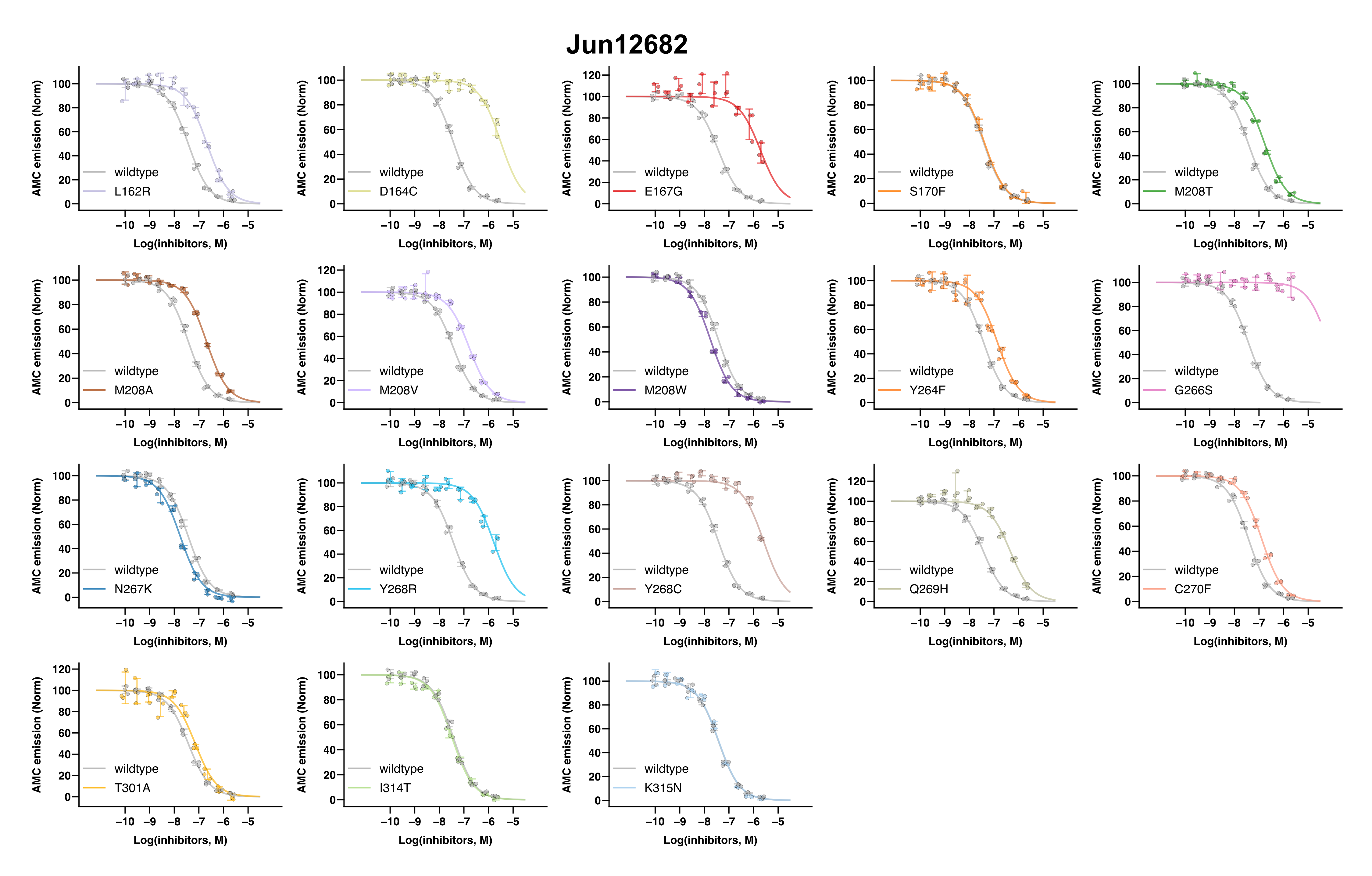

Supplement: S14 Fig — In each panel, the emission from buffer-only control (no PLpro) was normalized 0, and the emission from samples without inhibitor was normalized to 100. Inhibitor concentrations are plotted on a logarithmic scale on the x-axis, with normalized emission on the y-axis. (TIFF) [file ppat.1013468.s014.tiff]

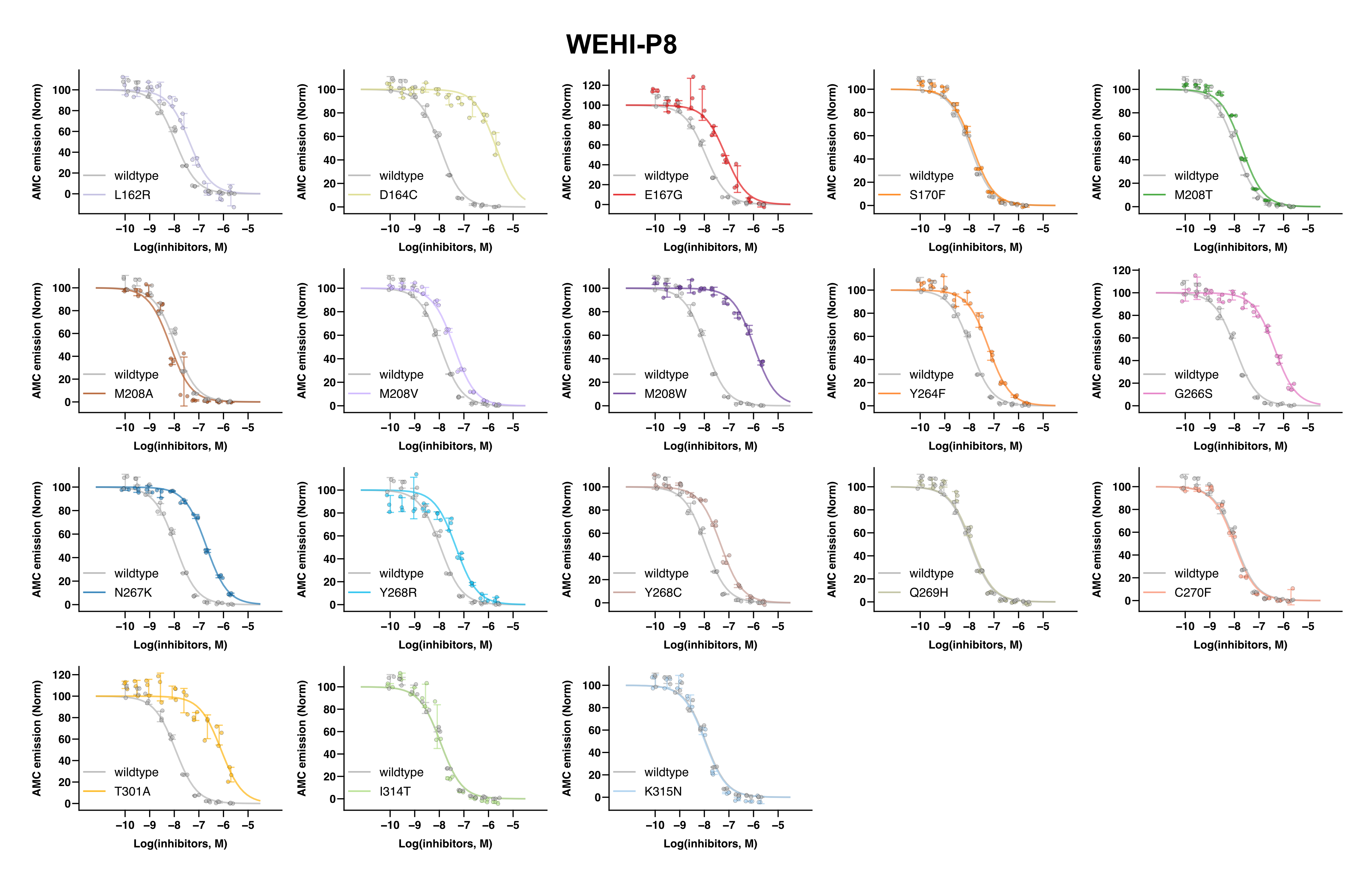

Supplement: S15 Fig — In each panel, the emission from buffer-only control (no PLpro) was normalized 0, and the emission from samples without inhibitor was normalized to 100. Inhibitor concentrations are plotted on a logarithmic scale on the x-axis, with normalized emission on the y-axis. (TIFF) [file ppat.1013468.s015.tiff]

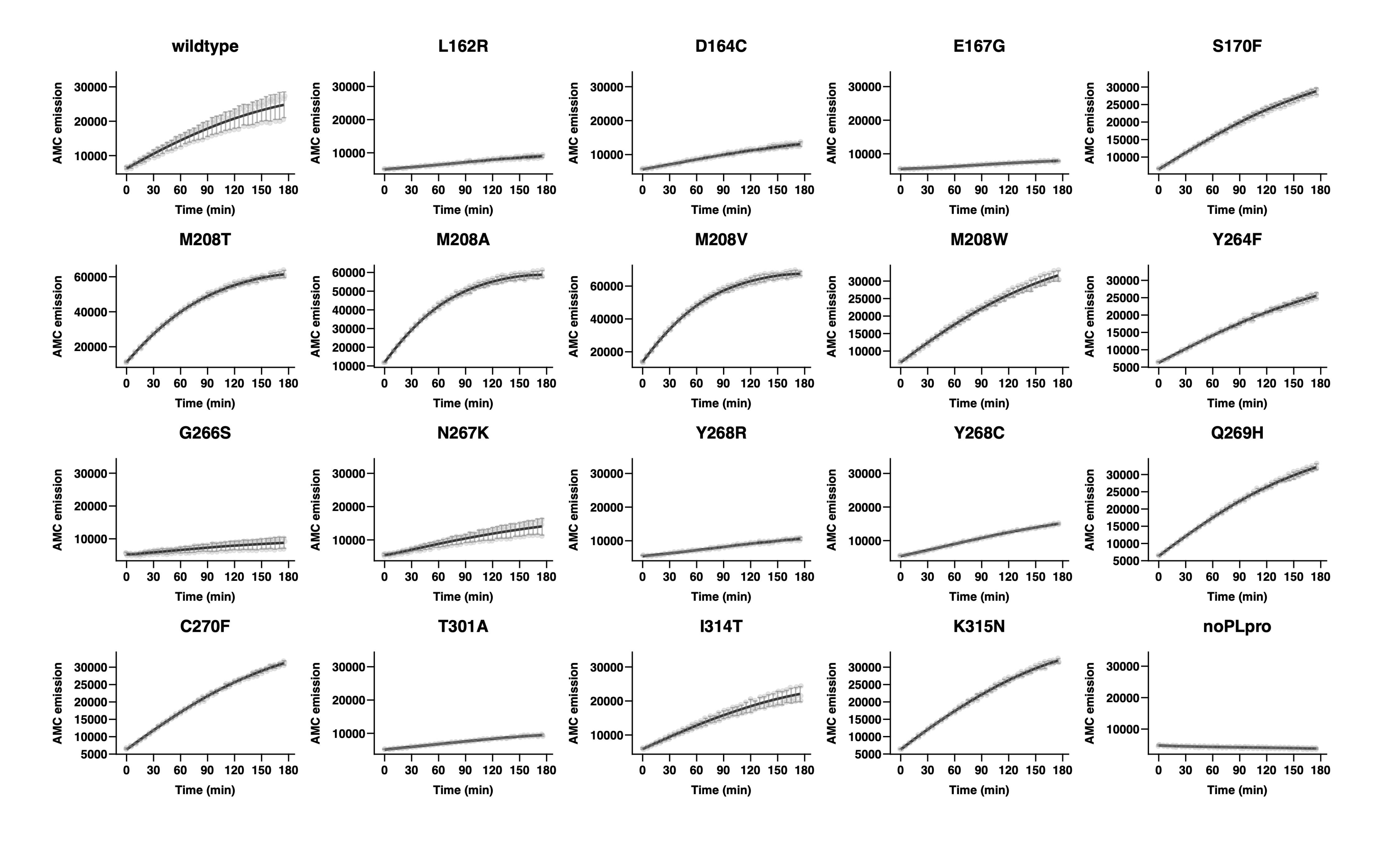

Supplement: S16 Fig — AMC fluorescence was measured every 5 minutes for 3 hours. Each dot is an individual measurement, while the curve represents the mean of three technical replicates from one representative experiment. Similar results are observed across two independent biological replicates. (TIFF) [file ppat.1013468.s016.tiff]

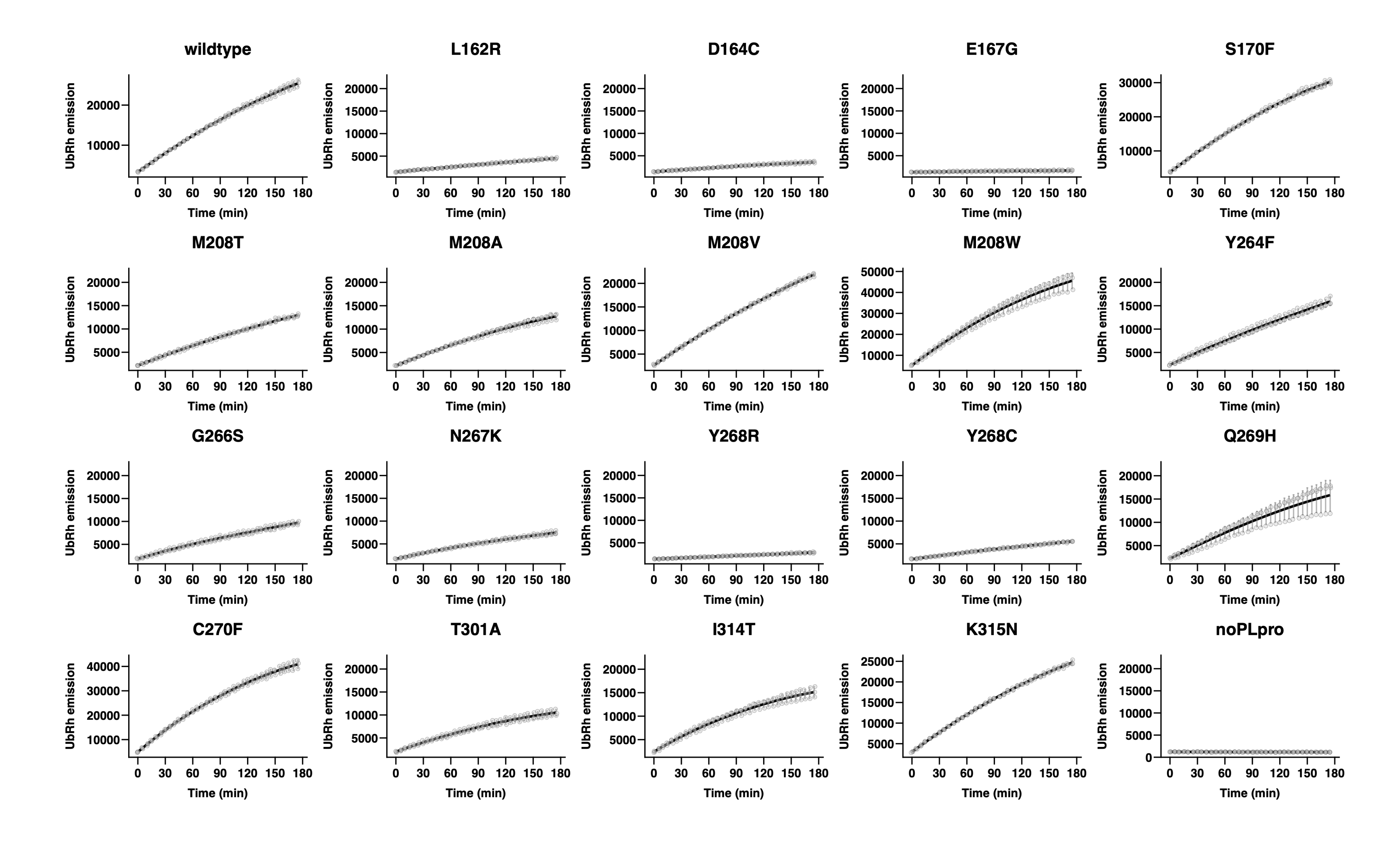

Supplement: S17 Fig — Rh110Gly fluorescence was measured every 5 minutes for 3 hours. Each dot is an individual measurement, while the curve represents the mean of three technical replicates from one representative experiment. Similar results are observed across two independent biological replicates. (TIFF) [file ppat.1013468.s017.tiff]

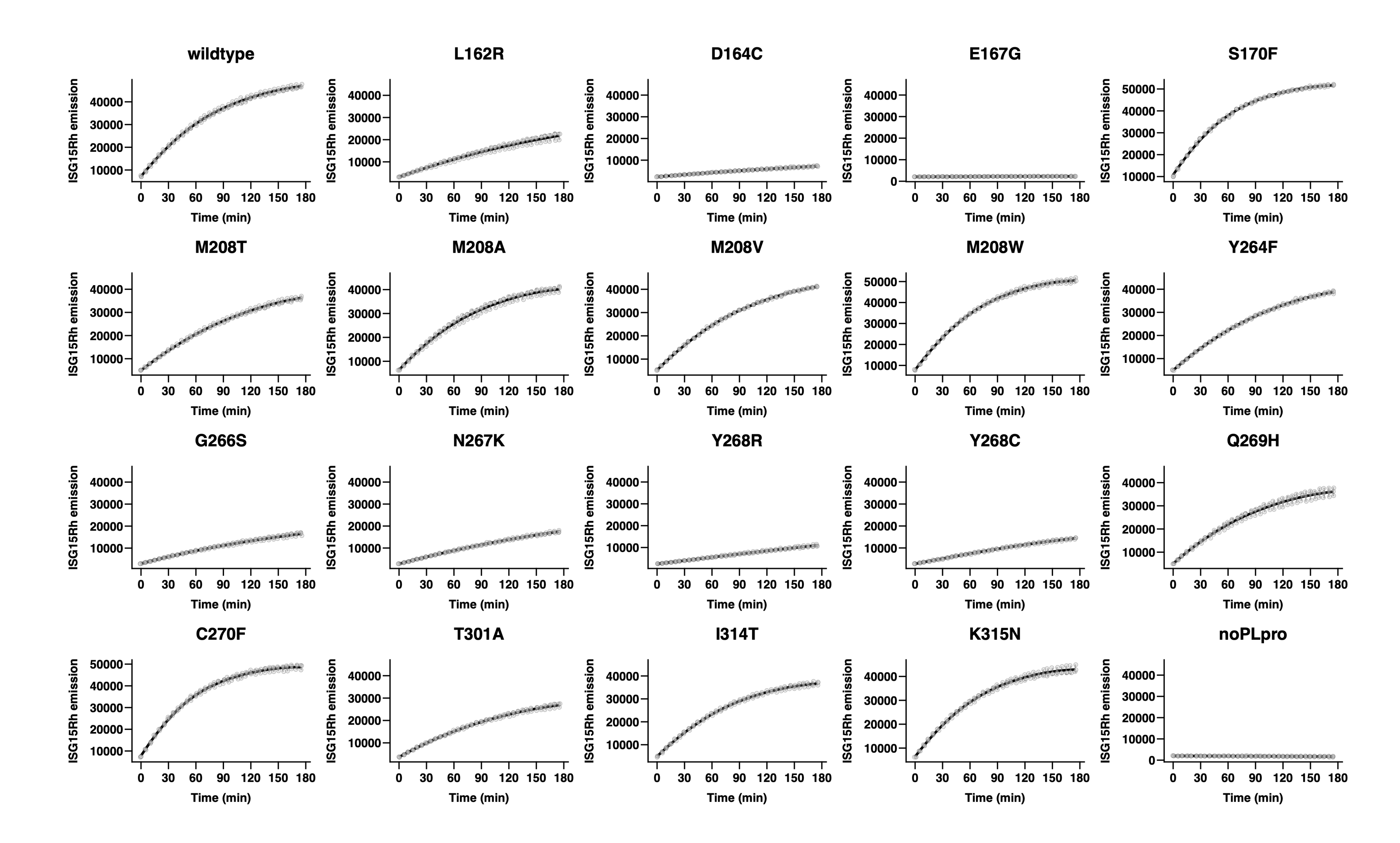

Supplement: S18 Fig — Rh110Gly fluorescence was measured every 5 minutes for 3 hours. Each dot is an individual measurement, while the curve represents the mean of three technical replicates from one representative experiment. Similar results are observed across two independent biological replicates. (TIFF) [file ppat.1013468.s018.tiff]

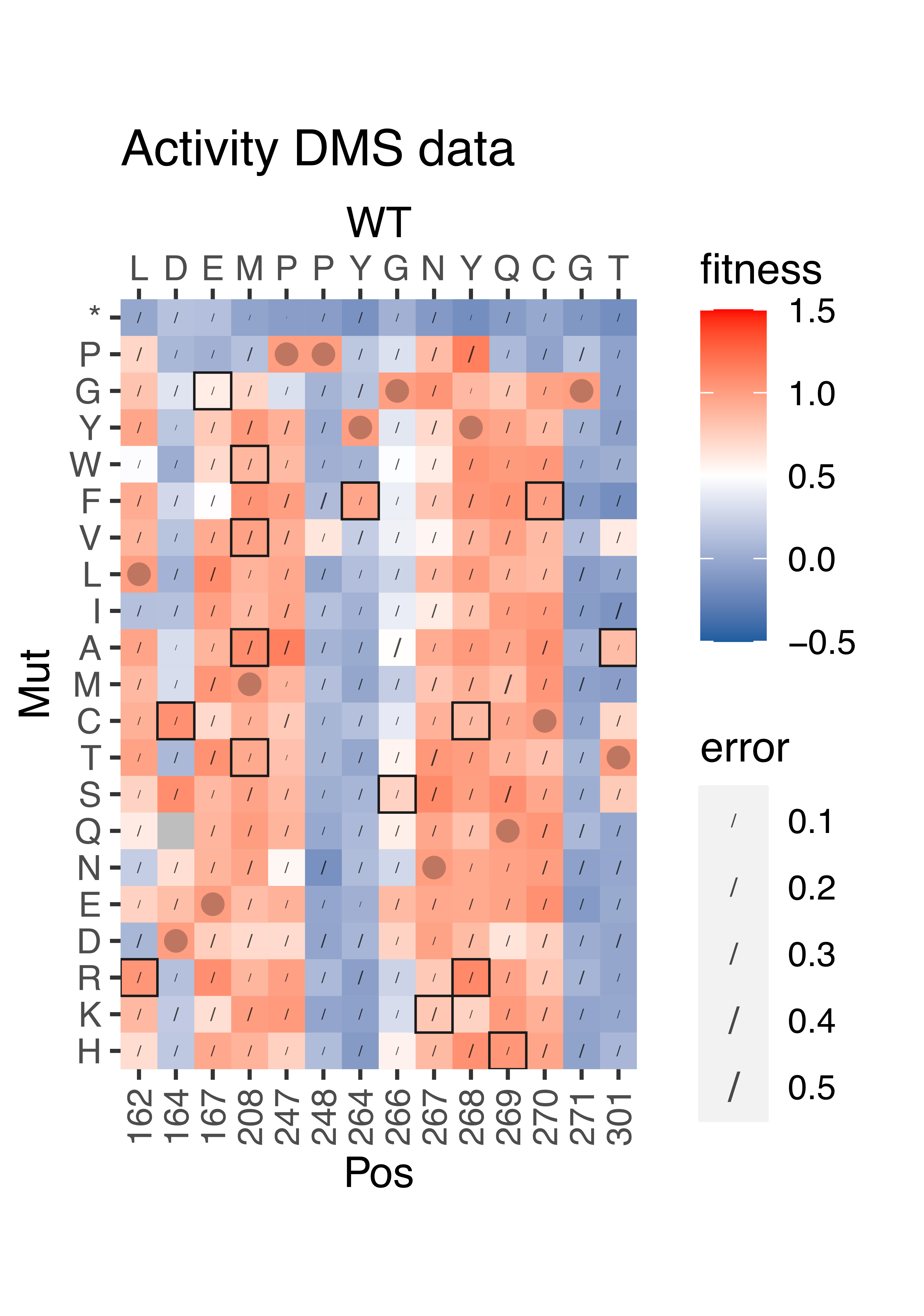

Supplement: S19 Fig — This sequence-function map snippet is of previously reported activity scores [18]. Variants are arranged with residue number on the x-axis and mutation type on the y-axis. The color scale represents the fitness scores for each variant. The means of wildtype and nonsense variants (residue 1–305) are normalized to 1 and 0, respectively. Each square corresponds to a single-residue substitution and includes an inset slash whose length is proportional to the estimated error. Wildtype residues are highlighted with a solid circle. Variants tested in our recombinant protein assays are boxed. (TIFF) [file ppat.1013468.s019.tiff]

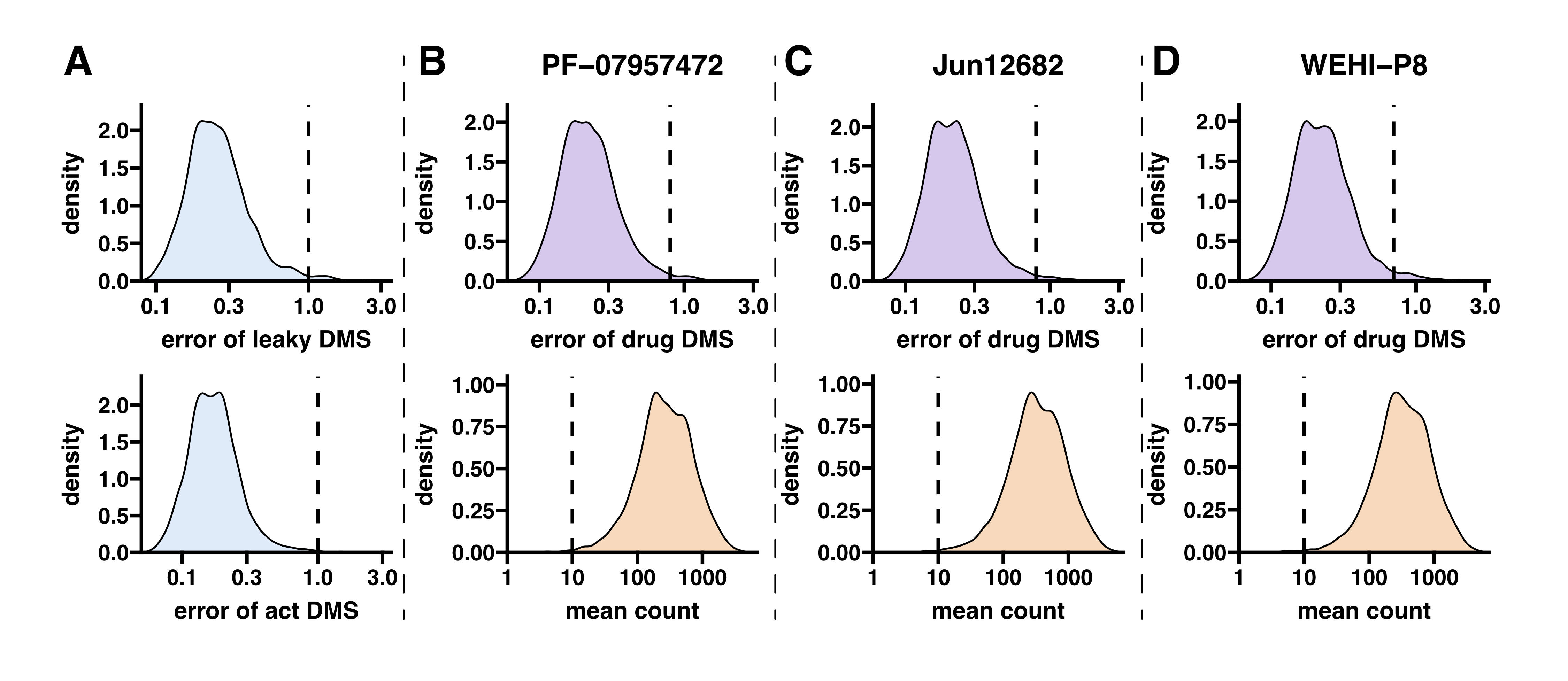

Supplement: S20 Fig — A) The distribution of variants with their errors from leaky dataset (top) and activity dataset (bottom) in the absence of inhibition. A cutoff at 1.0 was chosen based on the distribution profile and used to exclude variants from Venn diagrams in Fig 4F and 4G and mark variants with an X in S8–S10 Figs. B-D) The error (top) and mean count (bottom) distribution of PF-07957472 (B), Jun12682 (C), and WEHI-P8 (D). Cutoffs at 0.8 (error) and 10 (mean count) were used to exclude variants from Venn diagrams in Fig 4F and 4G and mark variants with an X in S8–S10 Figs. (TIFF) [file ppat.1013468.s020.tiff]

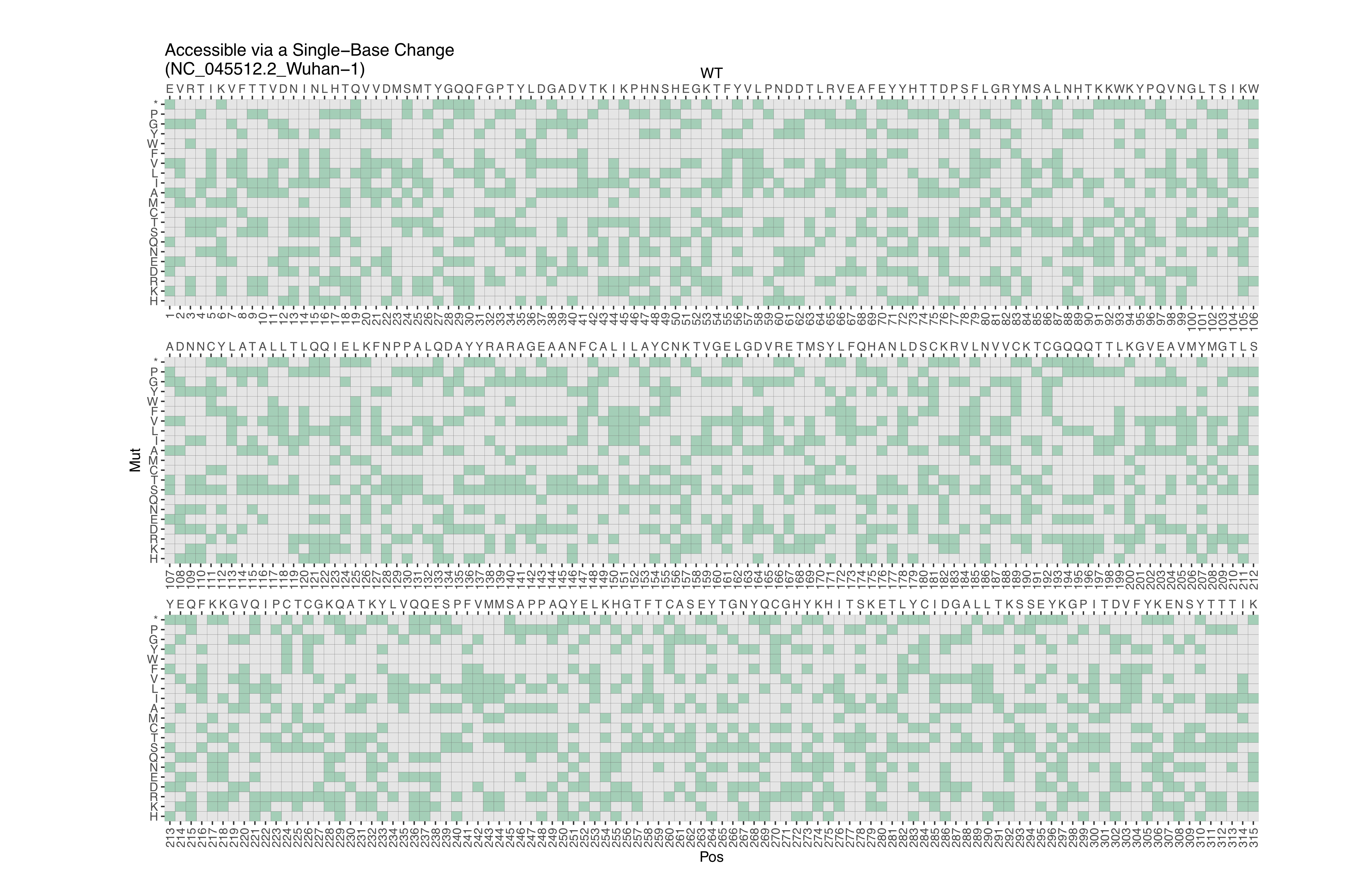

Supplement: S21 Fig — Wildtype PLpro sequence from the Wuhan strain (Genebank access number: NC_045512.2) was used in the analysis. Variants are arranged with residue number as x-coordinates and mutation type as y-coordination. Variants able to be achieved via a single base-pair edit are shown in green. Variants accessible by more than one base-pair mutation are shown in grey. (TIFF) [file ppat.1013468.s021.tiff]

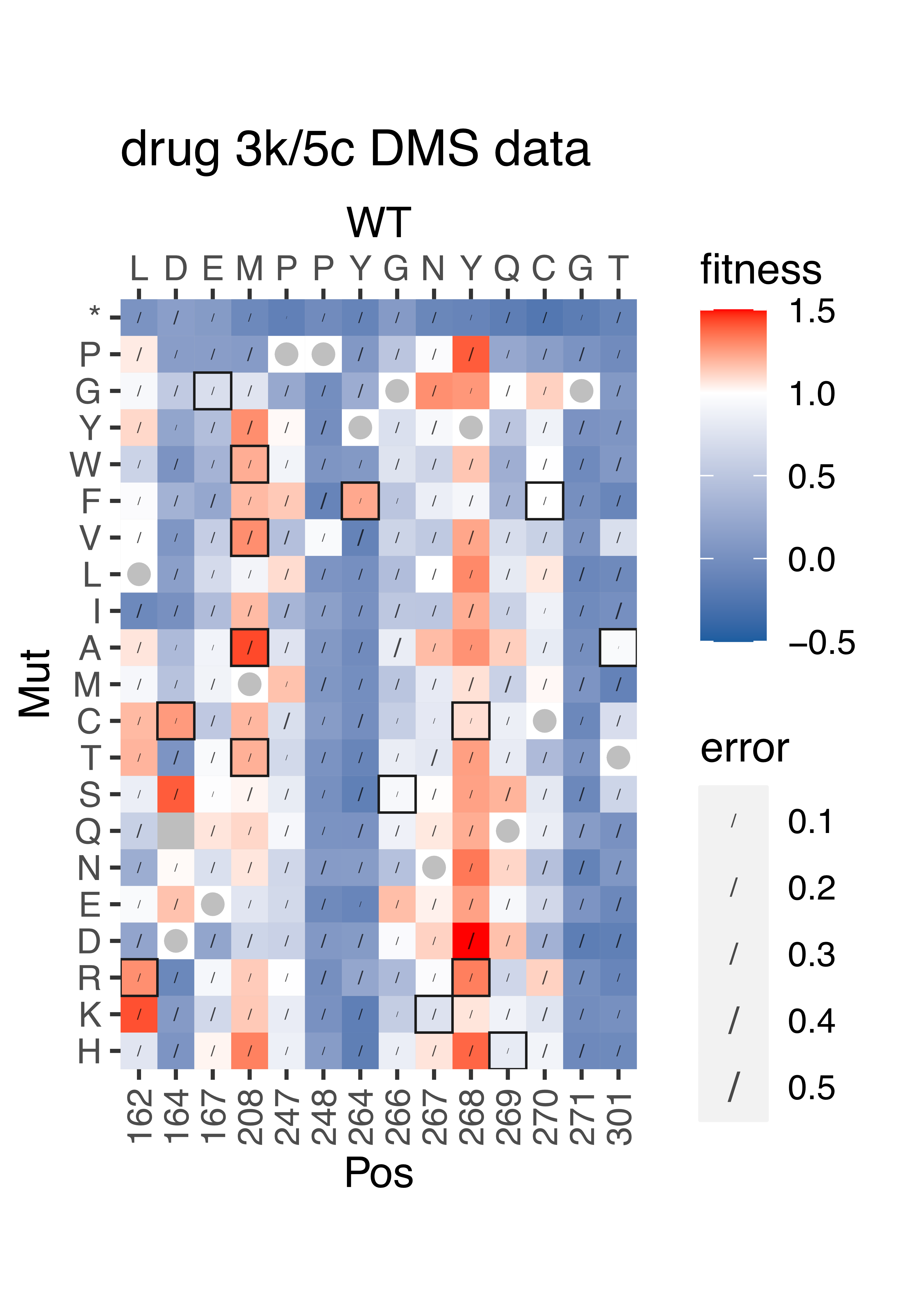

Supplement: S22 Fig — The data is downloaded from our previous publication [18]. Variants are arranged with residue number on the x-axis and mutation type on the y-axis. The color scale (shown in the color bar) represents the fitness scores for each variant. Each square corresponds to a single-residue substitution and includes an inset slash whose length is proportional to the estimated error. Wildtype residues are highlighted with a solid circle. The means of wildtype and nonsense variants (residue 1–305) are normalized to 0 and 1 respectively to enable easier interpretation. Those analyzed in the current study are boxed. (TIFF) [file ppat.1013468.s022.tiff]
