# Supplementary material for: Divergent resistance pathways amongst SARS-CoV-2 PLpro inhibitors highlight the need for scaffold diversity
Source: PLoS Pathog. 2025 Sep 5;21(9):e1013468. doi: 10.1371/journal.ppat.1013468 (PMC12431669; doi:10.1371/journal.ppat.1013468)
Supplement: Attachment — Submitted filename: Reviews and Responses PLoS Pathogens.docx [file ppat.1013468.s027.docx]

**Responses to Reviewers**

**Reviewer #1:**

Since the study includes a large number of mutational measurements, it may be worthwhile to consider some sort of approach to account for multiple tests (FDR, Bonferroni, etc). It is clear to me from the data that this is in no way going to change the conclusions in a meaningful way, and if not already implemented would make improve the statistical analyses. This is a minor issue and in no way detracts from my enthusiastic support of publication of this work in PLoS Pathogens.

*Thank you for the suggestion. We performed additional statistical analyses of drug escape variants by calculating Benjamini–Hochberg FDR‑adjusted q‑values to estimate the false‑discovery rate for escape events. This analysis indicated that a Z‑score of 4 was required before the FDR dropped below 0.05. However, our data exhibit positional structure, where conservative mutations at the same site tend to produce similar scores. For several positions (e.g., D164), we observe clear biological signal even for Z‑scores between 2 and 3. Excluding such positions would substantially underestimate the number of true escape variants, and as such we have not included this analysis in the manuscript. The appropriate statistical treatment of DMS data remains an evolving challenge. For example, a recent study by Coyote‑Maestas and Pimentel^1^ acknowledges the limitations of Benjamini–Hochberg FDR for DMS data and describes a Bayesian framework for controlling FDR in growth‑based DMS assays that incorporates positional information. While our use of multi‑dimensional DMS complicates direct application of this method, this study underscores that the field is still working toward robust statistical models for analysing DMS data.*

*1.	Rao J, Xin R, Macdonald C, Howard MK, Estevam GO, Yee SW, Wang M, Fraser JS, Coyote-Maestas W, Pimentel H. Rosace: a robust deep mutational scanning analysis framework employing position and mean-variance shrinkage. Genome Biol. 2024 May 24;25(1):138. doi: 10.1186/s13059-024-03279-7. PMID: 38789982; PMCID: PMC11127319.*

**Reviewer #2:**

The drug resistance of Jun12682 and PF-07957472 was recently reported (listed below). The authors are encouraged to compare the results from the current study and the results in the following paper. Tan, H., Zhang, Q., Georgiou, K. et al. Identification of naturally occurring drug-resistant mutations of SARS-CoV-2 papain-like protease. Nat Commun 16, 4548 (2025).

*We thank the reviewer for bringing this paper to our attention and for their constructive suggestions. We have added significant new text, analysis and figures to our manuscript to address the comments and queries below and we feel that this has greatly strengthened the rigour and potential impact of our study.*

*The recent publication from Tan et al provides a beautiful validation and extension of some of our results using a completely different approach, making the two papers highly complementary. We have incorporated a significant new section in our Discussion (p22) highlighting the complementary findings from these two studies.*

***The relationship between drug escape and viral fitness***

*A study published while this work was under review has directly examined the question of whether PL^pro^ mutations that confer drug resistance are fully compatible with viral replication and therefore likely to gain traction in widespread circulation. Using a structure-guided, candidate-based approach to select and test naturally occurring variants likely to escape GRL0617-based inhibitors, Tan and Zhang et al also identified E167, Y268 and Q269 as primary drug-escape hotspots (32). They further showed by serial passage of SARS-CoV-2 virus in the presence of increasing amounts of Jun12682 that E167G and Q289H mutants emerged spontaneously and were equally cross-resistant to PF-07957472. This result underscores the significance of our data showing that WEHI-P8 retains significant activity against E167G and full activity against Q269H. These mutant viruses exhibited mildly attenuated replication rates, consistent with the observation in their study and in ours that the enzymatic activities of E167G and Q269H mutant PLpro recombinant proteins are slightly reduced. Interestingly, second-site mutations at V98, L152 and T168 also emerged from the serial passage experiments that significantly enhanced viral fitness in the presence of Jun12682 even though these mutations do not independently confer detectable drug resistance, which our data also confirm. This highlights the reality that even mild fitness costs associated with drug resistance can select for compensatory mutations mitigating those effects, and it suggests that further DMS screening of second-site mutations on the background of the strongest drug resistance mutations may also have significant predictive value. Finally, as we reported in a previous study (18) and extended in the experiments described here, Tan and Zhang et al also identified that some M208 variants confer increased enzymatic activity and mildly affected inhibition by GRL0617-based compounds. The strong agreement in results between their candidate-based approach and our unbiased screening approach makes these two studies highly complementary and bolsters confidence in the real-world relevance of the unique WEHI-P8 resistance profile.*

One critical aspect of drug resistance is the impact of mutations on viral replication fitness. Although the authors have not validated the identified PLpro mutations using recombinant viruses, a detailed discussion of the enzymatic activity of these mutants—and how they compare to the wild-type enzyme—is warranted. Such analysis would provide important insights into the functional consequences of resistance mutations and their potential to emerge and persist in circulating viral populations. More importantly, the authors should also discuss which PLpro mutations are most likely to arise naturally based on mutation frequency, structural constraints, and evolutionary conservation.

*We have added panels to Figure 4 to show fitness scores for variants observed in sequencing data of circulating variants deposited on GISAID (4D). Many of the variants identified as potential escape variants have previously been observed in circulating viruses (4I). Together these new panels allow readers to judge the likelihood of an escape variant to arise naturally. In Dataset S1 we also add columns with GISAID observations and whether a given variant is accessible via single-nucleotide substitution, for all variants.*

*We have added text in the Results section (p14-15, 17) to discuss these findings:*

*"* *Whether lowered PLpro activity impacts viral fitness is not determined in our assays. However, millions of viral isolates have been sequenced throughout the course of the pandemic and this data can be mined to determine if any particular mutant has been observed as naturally occurring, and thus unlikely to be catastrophically detrimental to viral fitness. We downloaded the latest GISAID (31) data (July 23rd, 2025) and extracted reads that contained PLpro sequences, ignoring partial reads and those with indels. 16,083,184 sequences passed our filters, with 12,701,796 corresponding to the canonical PLpro sequence upon which our library is based. 3,074,197 contained a single amino acid (aa) change, 292,924 had double aa substitutions, 13,604 had triple aa substitutions and only 682 sequences contained more than three mutations. Among the 17 protein variants we tested in isolation, E167G, S170F, M208T, M208V, G266S, N267K, Y268C, Q269H, C270F, T301A, I314T and K315N have been observed in patient isolates (Fig 4C, S2 Dataset). S170F, M208T and M208V, while still rare in the context of the sequences reported, were more frequently observed compared to the other variants. E167G, the variant that had the lowest activity against all three substrates, had been observed 103 times. While activity over 30 minutes in biochemical assays is very slow for this variant, in cells it is able to cleave a significant proportion of biosensor, with a fitness score of 0.59, where 1 is active and 0 is inactive (S19 Fig) (18). The observation that this variant is present in circulating virus, albeit with reduced catalytic efficiency, indicates that even slow proteolysis may be able to support viral replication. The lowest activity variant as measured by our cellular assay that supports escape from PF-07957472 was G266Y with a fitness score of 0.365 and an associated error of 0.077.*

*To determine if low activity variants are permissible in the context of viral fitness, we stratified the number of observations from the GISAID dataset and plotted fitness scores for variants that are accessible by a single base pair change that were: absent from the dataset, observed 1-10 times, 10-100 times and so forth, to visualize the spread of activities at different observation rates (Fig 4D). We saw strong enrichment of functionally impaired variants in the bin containing variants that were absent from the GISAID dataset, indicating that most single base pair substituted variants that support function have been observed. Sequencing errors may account for many observations; however, we saw strong enrichment for active variants starting in the gate with 10-100 observations. Poorly active variants were found even in the 1,000-10,000 observation bin but were absent at higher counts. While it is difficult to delineate an absolute activity threshold required for viral fitness from these data, it is likely that poor proteolytic activity can be overcome by the virus in the context of the extended timeframes of natural infection coupled with the proximity of covalently linked substrates during polyprotein cleavage. When the virus is under selective pressure, second and even third site mutations have been observed that likely act to restore activity to variants that escape selection pressure at the cost of fitness (32). Together these data provide a framework for discerning variants that drive drug escape from those that stabilize or enhance enzyme structure and function."*

*...*

*"* *Finally, we plotted the Z-score for each escape variant against the number of observations in GISAID data to gauge the likelihood of resistance emerging from each compound (Fig 4I). Results showed every escape variant that was accessible by a single base pair mutation (except Y264F) had been seen at least once. No escape variant that required two or more base pair changes was observed except D164S, which can escape all three compounds and has been observed 3 times. We provide a table of all predicted escape variants, their Z-score, activity fitness scores, accessibility with only one base pair change and the number of times they were observed in GISAID data (S2 Table)."*

Can the cellular FRET biosensor assay detect PLpro variants carrying double or triple mutations? It is well established that resistant viruses selected through serial viral passage often harbor multiple mutations.

*The FRET biosensor assay can detect significantly resistant variants regardless of their mutational burden, but our library contained no multi-site variants by design, because the combinatorial space rapidly becomes too large to adequately survey in a comprehensive and unbiased manner. Interestingly, the Tan et al study identified several examples of second-site mutations that increased the drug resistance of E167G and Q269H mutants – specifically V98L, L152F and T168I – and our screen does provide independent verification that these mutations do not independently confer resistance. We now highlight this point specifically in our new Discussion section, lines 505-508.*

Furthermore, have the authors evaluated the enzymatic activity or resistance profile of any double or triple mutants derived from the identified single mutations? Such analysis would provide a more comprehensive understanding of potential resistance pathways.

*We have not tested combinations of mutants identified in our screen. While we feel this is beyond the scope of the present study, it is a valuable suggestion and something that could be done in a future study, for example by constructing second-site variant libraries on the background of the two major resistance mutations that emerged in the Tan et al serial passage experiments. We also highlight the potential value of such a study in the new Discussion section, lines 508-512.*
